# Better Indigenous Risk stratification for Cardiac Health study (BIRCH) protocol: rationale and design of a cross-sectional and prospective cohort study to identify novel cardiovascular risk indicators in Aboriginal Australian and Torres Strait Islander adults

**DOI:** 10.1186/s12872-017-0662-7

**Published:** 2017-08-23

**Authors:** Marc G. W. Rémond, Simon Stewart, Melinda J. Carrington, Thomas H. Marwick, Bronwyn A. Kingwell, Peter Meikle, Darren O’Brien, Nathaniel S. Marshall, Graeme P. Maguire

**Affiliations:** 1 Baker Heart and Diabetes Institute, PO Box 6492, Melbourne, VIC 3004 Australia; 2 Mary MacKillop Institute for Health Research, Australian Catholic University, Melbourne, VIC Australia; 3 The Woolcock Institute of Medical Research, The University of Sydney, Sydney, NSW Australia; 4 Sydney Nursing School, The University of Sydney, Sydney, NSW Australia

**Keywords:** Cardiovascular disease, Risk stratification, Aboriginal, Torres Strait islander, Indigenous, Echocardiography, Lipidomics, Sleep

## Abstract

**Background:**

Of the estimated 10–11 year life expectancy gap between Indigenous (Aboriginal and Torres Strait Islander people) and non-Indigenous Australians, approximately one quarter is attributable to cardiovascular disease (CVD). Risk prediction of CVD is imperfect, but particularly limited for Indigenous Australians. The BIRCH (Better Indigenous Risk stratification for Cardiac Health) project aims to identify and assess existing and novel markers of early disease and risk in Indigenous Australians to optimise health outcomes in this disadvantaged population. It further aims to determine whether these markers are relevant in non-Indigenous Australians.

**Methods/design:**

BIRCH is a cross-sectional and prospective cohort study of Indigenous and non-Indigenous Australian adults (≥ 18 years) living in remote, regional and urban locations. Participants will be assessed for CVD risk factors, left ventricular mass and strain via echocardiography, sleep disordered breathing and quality via home-based polysomnography or actigraphy respectively, and plasma lipidomic profiles via mass spectrometry. Outcome data will comprise CVD events and death over a period of five years.

**Discussion:**

Results of BIRCH may increase understanding regarding the factors underlying the increased burden of CVD in Indigenous Australians in this setting. Further, it may identify novel markers of early disease and risk to inform the development of more accurate prediction equations. Better identification of at-risk individuals will promote more effective primary and secondary preventive initiatives to reduce Indigenous Australian health disadvantage.

## Background

Aboriginal Australian and/or Torres Strait Islander peoples (Indigenous Australians) have well-documented health disadvantage in comparison with non-Indigenous Australians [1]. Addressing health disparities in an Australian Indigenous setting ultimately rests on the development and implementation of primordial and primary prevention strategies to address population and individual risk factors that facilitate disease development. In the interim, secondary prevention strategies are required to identify and manage people with established disease to prevent progression and complications.

There is an estimated 10–11 year gap between Indigenous and non-Indigenous Australian life expectancy at birth and, of this, one quarter is attributable to cardiovascular disease (CVD) [2]. Importantly, the pattern and natural history of CVD that presents in Indigenous communities is quite different to that seen in other Australian communities [3, 4]. For example, a recent study in central Australia [5] reported that 5% of a sample of Aboriginal adults had evidence of heart failure, up to four times the reported prevalence of heart failure in the overall Australian population [6], with an onset at an earlier age.

There is, therefore, a strong rationale to develop more culturally and contextually appropriate strategies to address the disparity in life expectancy between Indigenous and non-Indigenous Australians resulting from CVD. In particular, improvements in the primary and secondary prevention of CVDs in Indigenous Australians has the potential to significantly reduce this gap. This paper describes the BIRCH (Better Indigenous Risk stratification for Cardiac Health) project that aims to assess cardiovascular risk and explore the potential value of novel cardiovascular risk factors and early disease markers in Indigenous Australians. The objective is to more accurately identify Indigenous Australians at risk of overt CVD so that preventive treatments can be commenced sooner in those whom will receive the greatest benefit. More specifically, BIRCH will determine whether measures of left ventricular dysfunction, sleep quality and sleep disturbances, and detailed lipid profiles are useful in determining the risk of development of overt CVD in Indigenous Australians such that interventions can be commenced to prevent or retard its development. In addition, it will seek to determine whether there is any difference in the utility of such measures between rural and urban Indigenous populations and whether such measures may have relevance in non-Indigenous Australians.

### Traditional cardiovascular risk stratification and Indigenous Australians

A number of CVD risk stratification scores have been developed to identify individuals at risk of CVD who may benefit from the early introduction of preventive therapies [7, 8]. One of the most extensively used prediction algorithms is the Framingham Risk Score derived from the Framingham Heart Study [9, 10]. While the risk factors identified in this and subsequent Framingham studies [11] (age, sex, high blood total cholesterol, low high-density lipoprotein (HDL) cholesterol level, high systolic blood pressure, the presence of diabetes, and smoking) have important utility in non-Indigenous populations, research suggests that their predictive power in Indigenous Australian populations is limited. For example, the research of Wang and colleagues in remote Aboriginal Australian communities demonstrated significant discrepancies between the predicted incidence of coronary heart disease (CHD) based on the Framingham score (4.4/1000 person-years) and observed incidence (11.0/1000 person-years), with the greatest discrepancy occurring in younger women in whom the observed rate of CHD was over 30 times the predicted rate [12, 13].

Such findings suggest that additional factors not included in the Framingham functions may be important in adding to, or interacting with, traditional CVD risk factors to result in the increased and currently ‘unmeasured’ risk of CVD in Indigenous Australians. For example, Wang and colleagues demonstrated that waist circumference, body mass index (BMI), hip circumference, and high sensitivity C-reactive protein (hs-CRP) were significantly associated with cardiovascular risk independent of other cardiovascular risk factors [14, 15]. Similarly, McDermott and McCulloch found that diabetes in Aboriginal Australians in remote north Queensland, particularly when associated with albuminuria, was associated with a far higher risk of CHD compared with traditional Framingham risk factors [16], while Luke and colleagues identified hyperglycaemia (fasting glucose > 4.8 mmol/L) and albuminuria (albumin:creatinine ratio > 5.7 mg/mmol) as clinical predictors of primary CVD in Aboriginal Australians from central Australia [17].

These earlier studies predominantly or exclusively involved Indigenous Australians living in remote or outer regional Australian centres. Whether their findings are generalizable to Indigenous Australians residing in major cities and inner regional centres, where 57% of Indigenous Australians live [18], remains unclear. While national Australian data would suggest the disparity between Indigenous and non-Indigenous CVD incidence as measured by age standardised hospitalisation is far lower in urban as compared with remote residing Indigenous Australians [19], this is not reflected in single centre studies. In Bradshaw and colleagues’ follow-up of the Perth Aboriginal Atherosclerosis Risk Study (PAARS) cohort, the authors demonstrated city-based Aboriginal Australians had a rate of first CHD events (hospital admission or CHD death) that was far higher than city-residing non-Indigenous Australians [20] and comparable to Indigenous Australians residing in remote Northern Territory communities [21].

### Beyond traditional risk markers for CVD

Whilst many existing and newer markers of early CVD disease or risk may help identify Indigenous Australians at increased risk of development of overt CVD, they often do not independently provide new or additional targets for primary or secondary prevention. Furthermore, such markers continue to focus predominantly on atherosclerosis-mediated CVD despite the increasing recognition of non-atherosclerotic causes of conditions such as heart failure [22, 23]. We have therefore selected three particular areas of interest that may either increase the risk or be early markers of CVD and are potential targets for primary and/or secondary prevention in this setting.

#### Sleep quality and disorders

Fatigue and poor sleep quality have been found to be related to CVD risk factors including hypertension and CHD, attributable to increased sympathetic activity [24]. Poor sleep also predisposes to the metabolic syndrome [25] and mood disturbance [26]. Whilst the literature confirms poor sleep quality and duration as a risk factor for CVD, there is little understanding of the impact of sleep hygiene and sleep behaviours, as compared with formal sleep disorders, in disadvantaged populations at high CVD risk such as Indigenous Australians.

Specific sleep-related disorders such as obstructive sleep apnoea (OSA) are associated with an increased risk in the development and progression of CVD [27, 28]. One recent observational study demonstrated a significant correlation between OSA and the presence of CHD, pulmonary hypertension, congestive heart failure, cardiomyopathy, and arrhythmia [29]. It has also been suggested that OSA is a risk factor for stroke and stroke-related death [30–32]. Analysis of the Western Australian-based Busselton Health Study, primarily in Caucasians, has confirmed that the presence of OSA has additional prognostic significance for all-cause mortality and stroke incidence beyond the risk that is explained by factors included in the Framingham score [31]. The additional predictive ability of OSA in Indigenous Australians is presently unknown.

Despite the evidence of an association between sleep disorders/quality and CVD, little has been achieved in the management of sleep disruptions for Indigenous Australians. Indeed, one recent study demonstrated that regional and remote Australians residents accessed diagnostic sleep studies at approximately 1/20th of the overall Australian rate [33]. Despite this lack of access, this retrospective cohort study of 200 adults revealed that Indigenous Australians were almost twice as likely to have a sleep disorder diagnosed when a sleep study was performed compared with non-Indigenous patients.

#### Left ventricular function and morphology

Markers of left ventricular function and morphology (including size and mass) may represent additional markers of either CVD risk or the earliest signs of pre-clinical CVD. Left ventricular ejection fraction (LVEF) is one of the most commonly used measures of left ventricular systolic function. Whilst it has been shown to be a strong predictor of mortality in patients with heart failure, deteriorating LVEF is often a late marker of disease. On the contrary, patients with signs and symptoms of heart failure or who have subsequent CVD events may still exhibit preserved left ventricular systolic function [34, 35]. Whilst there has previously been a focus on heart failure with reduced ejection fraction (HFrEF), it is evident that heart failure with preserved LVEF (HFpEF) is also associated with high rates of morbidity and mortality [36]. A focus beyond LVEF in assessing left ventricular function is therefore required to better understand whether more subtle alternations in heart function may represent the earliest signs of CVD, particularly at a stage when treatment may reduce progression to overt CVD. Particular targets in this regard include global longitudinal strain (GLS) and measures of left ventricular morphology.

GLS is a novel index of myocardial deformation that can be quantified via automated speckle tracking echocardiography (STE). GLS has been validated against tagged magnetic resonance imaging and is more accurate than standard echocardiographic measurements of LVEF [37, 38]. GLS has been shown to more accurately predict cardiac events and all-cause mortality than LVEF in the general population and in patients with heart failure [37, 39, 40]. It may also be useful in the prognosis of individuals following myocardial infarction and cardiac surgery and in those with cardiomyopathy, aortic stenosis and chronic kidney disease [37, 38].

Other measures of left ventricular morphology, and particularly left ventricular mass and volume, may also represent a common endpoint for other cardiovascular risk factors including systolic blood pressure, body mass index, smoking and diabetes [41]. In addition, increasing left ventricular mass is associated with a greater risk of sudden cardiac death in patients with stable CHD [42] or uncomplicated essential hypertension [43, 44] and in the general population [45].

#### Lipidomic profiling

Lipidomic analysis permits the simultaneous quantification of several hundred lipid species within plasma, thereby providing a global assessment of lipid metabolism [46–48]. While Framingham cardiovascular risk assessment and broader cardiovascular assessment incorporate a small number of lipid profile elements (notably triglycerides, total cholesterol, HDL (high-density lipoprotein)-C and LDL (low-density lipoprotein)-C) it has been argued that these parameters only provide a very narrow snapshot of the dynamic processes of lipid metabolism [46].

Compared to conventional clinical assessments, lipidomic approaches not only enable the quantification of a far greater number of lipid classes and individual species within plasma (up to approximately 600 lipid species), but can also be applied to lipoprotein sub-fractions including LDL, HDL and very low-density lipoprotein (VLDL) giving greater mechanistic context. Given the pivotal role that lipids play in the pathophysiology of atherosclerotic CVD, new lipidomic technologies provide a way of identifying novel lipid species involved in the pathogenesis of cardiovascular disease and which have potential as biomarkers of future disease risk [46].

Using lipidomic profiling Meikle and colleagues undertook a targeted lipidomic analysis of 305 lipid species in plasma from 220 individuals who were either healthy, had stable CHD or unstable CHD [47]. Results indicated that individuals with atherosclerosis had altered lipid metabolism and that specific lipid profiles could be correlated with different stages of CHD (healthy, stable CHD, unstable CHD) [46, 47]. In more recent work, Meikle and colleagues identified plasma lipid species that are associated with future cardiovascular events and cardiovascular death in individuals with type 2 diabetes mellitus and demonstrated that a subset of these can be used to improve prediction of cardiovascular outcomes compared to currently used prediction tools [49]. More generally, Stegemann and colleagues demonstrated in a prospective population study that three lipid species isolated and measured via lipidomics profiling improved cardiovascular risk prediction over traditional risk factors [50]. Such studies indicate that lipidomic profiles may have value for prediction of CHD and CVD more generally. Furthermore, such detailed understanding of the lipidome is particularly important given the emergence of newer therapies that target pathways not modulated by existing pharmacotherapy including HDL and PCSK9-targeted therapies [51–53].

### Study aim and hypothesis

Given previous findings that traditional risk factors for CVD derived from non-Indigenous Australian population cohorts may only provide limited predictive power in Indigenous Australian and other similarly disadvantaged [54, 55] populations, it is timely to re-examine systems for the assessment of CVD risk or detection of pre-clinical CVD in greater detail in this setting. To do this we plan to undertake a combined cross-sectional and prospective study to investigate techniques for improved CVD risk assessment and early CVD detection. We will do this by using novel markers that may assist in the identification of who is at greatest risk of subsequently developing overt CVD and in turn would benefit from targeted primary and secondary preventive initiatives. In addition, we will seek to determine if such associations are demonstrated in both Indigenous and non-Indigenous Australians and, if restricted to Indigenous Australians, whether they act differently in those residing in remote as compared with urban locations.

**Fig. 1:**
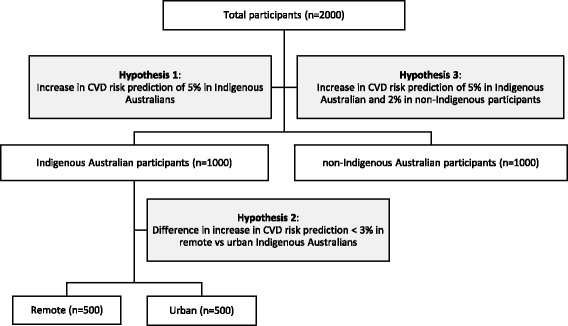
BIRCH research hypotheses and associated sample size required

Accordingly, in undertaking the BIRCH study, we hypothesise that markers of sleep quality/disorders, left ventricular function/morphology and select lipidomic species will:

1. explain more of the actual risk of the subsequent development of overt CVD in Indigenous Australian adults residing in remote Australia;
2. have equivalent additional explanatory power in Indigenous Australians residing in urban locations; and
3. capture some, but less, of the currently unexplained CVD risk in non-Indigenous Australians. (see Fig. 1)

It is anticipated the findings of this study and inclusion of these novel risk factors will provide evidence to inform the ongoing development of CVD risk detection, particularly in Indigenous and other vulnerable populations, and provide potential targets for the evaluation of interventions to enhance primary and secondary prevention of CVD.

## Methods/design

### Study design

BIRCH is a cross-sectional and prospective observational cohort study of Aboriginal and Torres Strait Islander (Indigenous) and non-Indigenous Australian adults (≥ 18 years) living in remote and urban Australia. It will adhere to STROBE guidelines [56]. BIRCH will adopt a three-phase approach that will reflect the hypotheses outlined above (see Fig. 1), first focusing on Indigenous Australians living in remote Australia, then urban-based Indigenous Australians and finally non-Indigenous Australians. Participants will be assessed for traditional CVD risk factors as well as potentially novel risk factors comprising sleep quality/disorders, detailed lipid profiles and markers of pre-clinical CVD including left ventricular function and morphology. Outcome data will comprise CVD events and CVD-related and all-cause mortality over a period of five years.

### Sample size

Sample size estimations will reflect the three-phase recruitment outlined above and in Fig. 1 and assume a two-side alpha of 0.05 and power of 80%. The first phase will investigate the increased predictive power of the inclusion of novel markers for future CVD risk in Indigenous Australians residing in remote Australia. It will assume an anticipated calculated Framingham-based five-year absolute CVD risk of 2.6% in line with earlier studies in this setting [13]. It is hypothesised that the addition of these novel measures will increase by 5% the correct identification of those who go on to develop overt CVD such that the absolute explained risk will be 7.6% compared to the current identification of only 2.6% utilising Framingham calculations. Based on these assumptions 115 participants will be required to detect this difference. This first phase in remote-residing Indigenous Australians will be expanded to 500 to take account of a potential failure to link all participants to administrative data sets relating to mortality and hospital separation, the exclusion of participants found to already have CVD at the time of enrolment, and the need for greater numbers to address subsequent hypotheses.

The second phase will investigate whether the additional predictive benefit of these novel markers is equivalent in remote and urban residing Indigenous Australians. It will hypothesise the anticipated Framingham-based five-year absolute risk of CVD of 2.6% is comparable in remote and city-based Indigenous participants. It will assume any difference in study-determined absolute risk for development of overt CVD (utilising novel measures) in the remote as compared with urban Indigenous cohorts will be less than 3%. Based on equivalence testing this would then require 738 Indigenous Australian participants, 369 remote and urban. Taking account of loss to follow-up and exclusion of those with pre-existing CVD this will be increased to 1000 in total with 500 remote and 500 urban Indigenous Australian participants.

The third and final phase of BIRCH will assume the additional predictive power of novel risk and disease markers will be less for non-Indigenous as compared with Indigenous Australian participants. It will assume the greater absolute risk of CVD explained by the addition of novel markers will be 2% in non-Indigenous Australian participants and, as for phase one, 5% for Indigenous Australian participants. To detect this difference will require 1176 participants, 588 Indigenous and 588 non-Indigenous participants. Given 1000 Indigenous Australian participants will be required for phase two of the study it is proposed a similar number of non-Indigenous Australian participants is recruited to address the highlighted issues above.

In summary the BIRCH study will anticipate enrolling a total of 2000 participants across three study phases including 500 Indigenous Australians residing in remote Australia, 500 Indigenous Australians residing in urban Australia and 1000 non-Indigenous Australians.

### Enrolling participants and informed consent

Potential participants will be recruited via a convenience sampling method. Whilst this will be an opportunistic community-based sample, we will aim to ensure that the gender and age distribution of the cohort will reflect that of the broader Indigenous and non-Indigenous Australian population [18].

Recruitment will take place with the assistance of local key informants with input from local research assistants and primary health care centres. In order to contact and enroll potential participants, BIRCH staff may visit the homes of such potential participants in the company of a local health care staff member or, where appropriate, a community-based Indigenous Australian research assistant.

Potential participants will be informed about the study using local media, community groups, Indigenous research staff and local language translators as required. Plain language patient information sheets will be provided and written informed consent obtained.

### Ethics

Participant recruitment and data collection for BIRCH will not commence at any site until approval has been granted by the relevant local human ethics research committees. The process of seeking ethics approval will necessarily involve consultation with local community, health and other stakeholders as well as consideration of local cultural issues.

### Data collection and outcomes

CVD risk assessment of participants will entail collection of data outlined in the Box below.

**Table:** 

• Demographic data • Family history of cardiovascular disease (self-report) • Co-morbidities and medication use (review of primary health care medical records and hospital separation data for the last ten years, current Pharmaceutical Benefits Scheme (Australian national medication subsidy scheme) dispensing history for urban-based participants) • Lifestyle behaviours – smoking, alcohol (self-report) • Clinical examination – blood pressure (Omron HEM-7130, Omron Corp., Kyoto, Japan), weight, height, waist/hip/neck circumference (standard anthropometric measurement methods) • Investigations - blood chemistry comprising HbA1c, total cholesterol, LDL-cholesterol, HDL-cholesterol, triglycerides, non-HDL-cholesterol, total cholesterol/HDL-cholesterol ratio. Blood chemistry will be measured from aliquots of venous blood collected from participants and analysed using an Alere Afinion™ AS100 Analyzer (Alere Inc., Waltham, MA, USA)	

At the time of initial assessment, participants will be provided with general feedback regarding their CVD risk profile based on existing Australian guidelines [57–61] and information regarding potential lifestyle changes that may reduce risk. If results of these initial investigations reveal any previously undetected chronic disease, then follow-up with local primary care centres will be facilitated.

In addition to collecting and communicating data regarding conventional clinical cardiovascular risk factors, the following investigations will be undertaken:

- Echocardiography with specific reference to left ventricular mass, function and strain studies (STE). This will reflect the approach of earlier studies as undertaken by Marwick and others [62]. Data will be collected using a standard operating procedure for image acquisition including two dimensional imaging, colour Doppler and pulse and continuous wave spectral Doppler analysis using a portable echocardiography platform (Vivid Q, GE Medical Systems, Milwaukee, WI, USA). Analysis of acquired data will be undertaken by a single core laboratory at Baker Heart and Diabetes Institute (Melbourne, VIC, Australia) using standardised analysis and reporting protocols.
- Screening home-based polysomnography (PSG) and/or actigraphy [63] for assessment of sleep disordered breathing (including obstructive sleep apnoea) and sleep quality respectively. PSG will include multichannel recording including oximetry, ECG, EEG, EMG, flow and chest and abdomen excursion using a small portable recorder (Alice PDx, Philips Respironics). A sleep scientist will attach equipment in the late afternoon and retrieve it in the morning. Polysomnography data will be analysed by a single core laboratory at the Woolcock Institute of Medical Research (The University of Sydney, NSW, Australia) using standardised analysis and reporting protocols. Actigraphy will involve participants wearing a device (ActiGraph wGT3X-BT, ActiGraph, Pensacola, FL, USA) attached to the waist for a minimum of 3 days that monitors sleep/wake patterns through recording of physical movement. Analysis of actigraphy recordings will be undertaken according to a standardised protocol by the same trained researcher at the MacKillop Institute, Australian Catholic University (Melbourne, Australia).
- Lipidomic analysis of both whole plasma and lipoprotein sub-fractions will be undertaken at Baker Heart and Diabetes Institute with an emphasis on HDL composition using electrospray-ionisation tandem mass spectrometry and HDL particle size and distribution using nuclear magnetic resonance (NMR) spectroscopy [47, 64].

If any of these specific investigations reveal a clinical relevant abnormality then research staff will make contact with participants and their local health care provider to facilitate appropriate follow-up.

Once these data have been collected and analysed, any participant found to have a pre-existing CVD based on primary care or hospital separation data will be excluded from further analysis and follow-up. The remaining participants will be stratified according to exposure. Exposure will be defined as the presence of conventional and potentially novel cardiovascular risk factors. Outcome data relating to CVD events and mortality over a five year follow-up period will be collected:

- Cardiovascular event data will be collected based on data linkage using ICD-10 [65] based hospital separation coding for CVD. This will included a diagnosis code range I20–I25 and procedure code blocks 669–679, reflecting the methodology of McDermott et al. [16].
- All cause and cardiovascular mortality will be ascertained using jurisdictional data and the National Death Index maintained by the Australian Institute of Health and Welfare.

### Blinding

Researchers involved in collecting and analyzing participant data under the four separate components of the BIRCH project (conventional risk factor assessment, echocardiography, PSG/actigraphy, lipidomics) will be blinded as to the participant’s assessment in other components of the study. Thus the researchers tasked with collecting data on conventional risk factors, the sonographers and cardiologist reporters involved in measuring and reporting left function and morphology via echocardiography, the technicians and sleep specialists responsible for reporting portable PSG/actigraphy, and the researchers tasked with undertaking lipidomics analysis will be blinded to results obtained by other researchers. Furthermore, the researchers involved in collection of outcome data will be blinded as to each participant’s baseline assessment.

### Data management

All data collected on paper-based forms will be forwarded to an independent data management unit at the MacKillop Institute for processing in REDCap (Research Electronic Data Capture) according to standard operating procedures. Reports from the echocardiogram, PSG, actigraphy and lipidomics investigations will be received in electronic format and integrated with the other personal and health related information. All electronic data will be stored in a password-protected folder on a network drive that is regularly archived.

Records will be de-identified and these non-identifiable data will be analysed using STATA version 14 (StataCorp, College Station, Tex, USA). No identifying information will be published or disseminated upon completion of the study.

Data will be stored for five years following the publication of research results or the end of study whichever is the later as per recommended guidelines [66].

### Statistical analysis

Data analysis will comprise descriptive statistics relating to previously detected and undetected CVD, the presence of traditional and potentially novel CVD risk factors, and assessment of CVD risk [10]. The relationship between traditional and potentially novel CVD risk factors will be investigated by correlational analysis controlling for relevant covariates. Subsequent data analysis will investigate factors independently associated with CVD events and mortality during a five year follow-up period. The influence of traditional and putative novel (echocardiographic, lipidomic and sleep) CVD factors will be determined using survival analysis with the log-rank test (Freedman method) and Cox proportional hazard modelling. As pre-existing cut-offs for novel risk factors do not exist, receiver operator characteristic analysis will be undertaken controlling for traditional risk factors. Appropriate cut-offs will be identified with a focus on the highest sensitivity for the presence or development of CVD. These cut-offs will be utilised in subsequent Cox proportional hazard modelling and a risk score developed using a derivation subset of the sample. The utility of this score in predicting CVD will validated using a subset of the sample and standard diagnostic validation techniques including sensitivity and specificity and positive and negative predictive value.

## Discussion

CVD risk assessment in disadvantaged populations, including Indigenous Australians, remains an ongoing challenge. Despite CVD being a major contributor to overall health disadvantage in Indigenous Australians, there exist significant deficiencies in currently used CVD prediction tools. This hinders the ability of health services to target and deliver primary prevention initiatives to those individuals at greatest need. Furthermore, the presence of increased CVD burden unexplained by traditional risk factor prediction also challenges the existing understanding regarding the mechanisms responsible for CVD development in this unique Indigenous Australian population.

In the context of this background, BIRCH aims to enhance the understanding of factors responsible for the greater burden of CVD disease in Indigenous Australians. While many existing and newer markers of early CVD disease or risk may help identify Indigenous Australians at increased risk of development of overt CVD, the BIRCH project specifically focuses on three markers comprising: sleep quality and disorders, left ventricular function and morphology, and select lipidomic species. These markers were chosen as they are potential targets for primary and/or secondary prevention in this setting. If results of the BIRCH study do indeed indicate that these markers are able to explain more of the actual risk of the subsequent development of overt CVD in Indigenous Australian adults then interventions are available that can be employed sooner to prevent or delay the onset of disease.

In turn, BIRCH will potentially provide information to inform the development of more accurate tools for CVD risk prediction as a means for improving the targeting of existing and emerging preventive therapies in those at greatest risk of heart disease, ensuring those who can gain the greatest benefit are identified and treated early. Finally, the BIRCH cohort will form a valuable future and ongoing resource to facilitate early evaluation of new treatments to prevent and manage CVD. Together these elements will serve to support the development of effective, appropriate and sustainable interventions to prevent progression and complications of CVD in Indigenous Australians, thereby addressing this significant contributor to health disparity.

## Abbreviations

BIRCH: Better Indigenous Risk stratification for Cardiac Health
CHD: coronary heart disease
CVD: cardiovascular disease
GLS: global longitudinal strain
HDL: high density lipoprotein
HFpEF: heart failure with preserved ejection fraction
HFrEF: heart failure with reduced ejection fraction
hs-CRP: high sensitivity C-reactive protein
LDL: low density lipoprotein
LVEF: left ventricular ejection fraction
NMR: nuclear magnetic resonance
OSA: obstructive sleep apnoea
PSG: polysomnography
STE: speckle tracking echocardiography
VLDL: very low density lipoprotein
